# Narrative Matters: Improving young people's mental health through neighbourhood initiatives – the role of ‘collective local intelligence’ in Manchester

**DOI:** 10.1111/camh.12766

**Published:** 2025-03-24

**Authors:** Joe Ravetz

**Affiliations:** ^1^ Manchester Urban Institute, School of Environment Education & Development Manchester University Oxford UK

**Keywords:** Environmental influences, social environment, community programmes, mental health, education, preventative intervention

## Abstract

**Background:**

Young persons’ well‐being is the key priority for this case study on the inner‐city neighbourhoods of Manchester, and the challenges of coordination and synergy between the many organizations involved.

**Methods:**

The ‘Local‐wise’ project draws from insights on ‘collective local intelligence’, and the methods of the ‘Pathways toolkit’ which can explore and work with stakeholders on ways forward.

**Results:**

The findings point towards positive linkages between pro‐active neighbourhood initiatives, and the young person's mental health/well being.

**Conclusions:**

There is huge potential for upstream preventive work, where ideally the local neighbourhood is a place of belonging, identity and livelihood, as a counter to the pressures of globalization, precarity and social media.

**Key Practitioner Message**

- Work in progress in inner‐city Manchester shows how neighbourhood based initiatives can help with young persons’ mental health and well‐being.
- This study applies the insights of ‘collective local intelligence’ for a system‐level perspective on urban transformation.
- There is huge potential for upstream preventive work on the neighbourhood as a place of belonging, identity and livelihood.

## Introduction

Much of the young persons' landscape is a source of stress and alienation, from social media, insecure jobs or unsafe streets and the CAMHS is notoriously under‐funded and fragmented. The Lancet Commission proposes ‘to identify, reverse, and mitigate harmful political, economic, and social policies that are undermining mental health and wellbeing… in young people’ (Lancet Psychiatry, 2024): very broad goals, which somehow have to find application at the local level.

Meanwhile, at this local level, there are countless civic initiatives and community‐based groups, with programmes on urban greening, creative arts, youth inclusion and safer streets. Are they enough for the crucial upstream promotion of mental health and well‐being – or could more be done with diminishing resources?

This brief overview takes three parts. First, we outline the case study of inner‐city Manchester, some leading local initiatives and the role of the ‘Local‐wise’ action research. Second, we draw on the theory and practice of ‘collective local intelligence’, the mutual learning and co‐production between a *wider* community with *deeper* layers of value (Ravetz, 2020), and its application to the young persons' agenda. Third, this suggests forward pathways, as explored by local stakeholders, for the upstream approach to young persons' mental health and well‐being.

## A young person's neighbourhood?

The case study of Hulme Moss Side and Rusholme (‘HMSR’) includes three inner‐city wards of Manchester, near the city centre and large health/education campuses. HMSR is a typical inner‐city area of mainly older terrace housing, with rapid gentrification, transient/student populations, high deprivation and over 120 languages spoken in local schools. Much of the urban environment crossed by major roads and unkempt streets, hazardous for pedestrians, fly‐tipping and general ‘urban disorder’. For young persons the data shows widespread anti‐social behaviour (ASB), periodic outbreaks of street crime, with a background of social media‐induced alienation.^1^ However, some areas also show strong community cohesion with many social initiatives – badged as the ‘passionate people’ of Moss Side – and most schools and colleges appear to work well in challenging situations. In response, Manchester City Council works through its ‘Teams Around the Neighbourhood’ (TAN):^2^ bringing together local government departments with providers of education, health, police, housing, transport and key community‐based services.

*Local‐wise* is a community‐based action‐research project, building on long‐term relationships with the TAN in this area.^3^ The project aim is challenging – how to do ‘more with less’, in an age of budget limits, structural challenges and rising expectations. The current priorities include three overlapping themes: (a) young persons' well‐being and crime prevention: (b) active travel and urban design: (c) ‘waste mismanagement’ (fly‐tipping) and the urban environment. For each, *Local‐wise* explores new pathways/platforms for coordination and synergy between many stakeholders, with the ‘participatory research’ approach and a conceptual framework for ‘collective local intelligence’, as in the next section (Ravetz, 2020).

### Local challenges: Stakeholder perceptions

On the young persons' theme, a wide range of inter‐connected challenges was explored through a programme of stakeholder workshops and interviews, using the Local‐wise pathway mapping method:

- *Social challenges*: mental syndromes are linked with general alienation/disempowerment. Safety/crime are linked with negative perceptions of police: substance/alcohol use and poor diet are linked with subcultures and global media.
- *Cultural challenges*: cultural exclusion and challenges for women/ethnic, and other minorities: lack of role and identity in society: combined with negative stereotypes and reputation of the area.
- *Environment challenges*: fly‐tipping, air pollution, urban disorder/degradation, general lack of green spaces and sports facilities in some areas.
- *Infrastructure challenges*: lack of safe, high‐quality transport and poor housing conditions, especially in the private rented sector.
- *Technology challenges*: overuse of social media can be addictive and alienating, especially for the most vulnerable.
- *Economic challenges*: young persons' poverty and financial dependency: with a real or perceived lack of secure, meaningful employment and career pathways.
- *Policy challenges*: general under‐funding and lack of public provision/investment: mental health provision is generally under‐funded, reactive and fragmented.

The overarching challenge was seen as social media, not only as potentially addictive and alienating but also as a globalized force that is often alien to older generations (Royal College of Psychiatrists, 2020). Problematic physical environments and exclusive social structures may then add to the pressure on young persons to live mainly online. Each of the above challenge areas was then mapped by stakeholders in terms of (a) organizations most involved, (b) positive visions to counter the challenge, (c) strategic pathway/road‐mapping for short‐/ medium‐/ long‐term action and (d) local initiatives in progress, as follows.

### Local initiatives

A cross‐section of local initiatives, programmes, stakeholders and organizations demonstrates the diversity of approaches to young persons' well‐being.

*Nursery Schools and Children's Centres* show some shining examples such as Martenscroft in Hulme. Here the general approach of ‘Family Hub’ comes alongside a local government service redesign of early years/early help, with initiatives such as Parent Carer Forums, all within the framework of the UNICEF Child Friendly City. Early Years Outreach Workers are on the front line of social cohesion, in effect acting as social workers/citizens' advisors, albeit with very limited resources. A key signal is a long‐term presence in the neighbourhood, where some former pupils now bring their own children back to the same school.

*Secondary schools* such as the Manchester Academy work by the ‘Cradle to Career’ framework, in the face of challenges with transient and/or non‐English speaking families, and long‐distance commuting for many pupils. There is a strong emphasis on the ‘Rights Respecting’ framework and the ‘personal curriculum’ for every pupil. However there are significant numbers in SEND categories and the CAMHS system, reporting long delays to assessment and treatment: there are some offsite provisions, but otherwise, the excluded then go to Pupil Referral Units. There are recurring problems with parental engagement, and in many cases, offsite meetings are better hosted by an independent CBO. For social media issues, phone use is banned within school hours, but evidence shows many pupils catching up just outside the school gate.

*Greater Manchester Police* is active in upstream prevention and community relations, with the new ‘Bee in the Loop’ platform.^4^ They provide direct funding where needed for ‘family‐youth’ groups, with a ‘community outreach’ offer, helping with youth clubs sports and practical bike projects with skills training. These are part of the ‘neighbourhood review’ model, which includes local profiles and stakeholders, with a focus on ‘hard to reach’ young persons. The Neighbourhood Policing model is set at the strategic level of the Combined Authority, with local PCSOs at the ‘Partners & Community Together’ stakeholder meetings. For young offenders, the ‘Another Chance’ panels consider low‐level offences with family issues and education/employment links: Meanwhile, Operation Encompass works closely with schools. Overall there is a challenge in young persons' often negative perceptions of the police, with implications for safety and mental well‐being, but current actions are pointing in positive directions.

*Housing conditions* are crucial to mental well‐being: while the local situation is mainly private‐rented and owner gentrification, some social landlords such as Mosscare St Vincent (MSV) are beacons in the. Their remit is to take tenants in bands 1–2 priority allocations, with an average waiting list time of 4–5 years. There is a strong outreach programme, including young persons' engagement groups, a dedicated ASB team and liaison with youth services for low‐level intervention. MSV is not a youth worker, but they carry a strong duty of care and safeguarding, as seen with joint NHS housing schemes and care‐leavers accommodation projects.

*Manchester City Council* sets a high priority on young persons' well‐being, with a new programme of Area Youth Forums.^5^ These aim to empower and enable young people by handing them the space, resources and initiative to run meetings, set agendas, act as ambassadors, signpost to resources, identify needs and ideas and allocate project funding (in limited amounts for the moment). Experience so far shows gaps and limits, as the young people most in need are often the least likely to join. The TAN officers then have a crucial role in building trust, maintaining relationships and empowering communities. In Hulme Park, for instance, the basketball facility now has 450 members: the local CBO ‘Hideaway’ does direct outreach to disaffected young persons: and Shaw Green estate is working on play streets and fly‐tipping problems via community‐based residents groups.

One shining example is the *Moss Side Powerhouse*, a multi‐service youth and community hub, hosting mental health, careers advice, NEET programmes, sports sessions, arts & crafts, youth clubs, music and drama sessions.^6^ The Powerhouse Library is one of the country's only dedicated youth libraries: NHS Emerge/16–17 CMHT is a multidisciplinary team offering mental health services in a community setting: Career Connect offers support into education, employment and training for young people: and Thrive in Education provides outreach mental health services.

## Towards a ‘Collective local youth intelligence’

While these initiatives are admirable, they should be seen in context and here the *Local‐wise* provides a relevant framework for ‘collective local youth intelligence’ (Ravetz, 2020). This is broadly, the mutual learning, synergy and co‐production between a *wider* community of interest (beyond the elite/experts): with *deeper* layers of value (beyond the functional/material): for *further* horizons of change (beyond the immediate problem). This framework then helps to explore the young person ‘nexus’ of inter‐connected challenges – de‐industrialization trauma, deracination of white males, myopic education systems, ‘depressive hedonism’ and social media dependency, as seen with the Manchester ‘youth landscape’ (Cavill Associates, 2024). With a co‐produced mapping of inter‐connected challenges, the *Local‐wise* stakeholders can then explore responses: creative visions, potential synergies and opportunities and likely pathways for action.

Such pathways aim to combine multiple approaches: creative futures and visioning, such as the ‘GM2040+’ local college project (Hawkins, Gardiner, & Ravetz, 2016): physical environment as both problem and opportunity (Ravetz, 2017): and the *Local‐wise* focus on institutions, networks and the ‘cognitive eco‐system’. In practical terms, this looks for potential ‘linkages’ (synergies, collaborations or other enabling measures) in four main dimensions:

- ‘Horizontal’ linkages between large service providers, such as NHS, GM Police, the city Council, schools and universities, transport, housing, etc.
- ‘Vertical’ linkages between central authorities and local service delivery, at neighbourhood, street or household levels.
- ‘Lateral’ linkages between service providers, local businesses and community‐based/third sector organizations, partnerships, networks and initiatives.
- ‘Citizen’ linkages at the street, household or individual level, where the primary focus is the inclusion and empowerment of young persons.

Each of these coordination agendas has two main tracks. The *Functional/online track* for information sharing, public access and technical analysis points towards digital solutions, remote monitoring, online platforms, interactive mapping and ‘smart city’ solutions. In parallel, a *People‐based track* aims for creative thinking, networking, problem/idea sharing, mobilizing projects and face‐to‐face working for social cohesion and community empowerment.

These coordination/synergy agendas are each component of a ‘collective local youth intelligence’, where potential synergies between individuals, households, communities and service providers are enabled by practical and creative action. Multiple approaches combine, such as local improvisation (Hamdi, 2004), service user empowerment (Cottam, 2018) or citizen organization (Alexander, 2022). For example, a Manchester park was a run‐down ‘problem area’ of weeds and stolen cars, until the residents began to set up festivals and markets, pop‐up youth clubs and graffiti walls – thereby building social cohesion, climate resilience and local livelihoods (Ravetz, 2020, 141–142). The implication is, the physical environment is not necessarily a fixed problem, more a space of creative potential.

## Next steps

For the structural challenge of young persons, there are few easy solutions, but many creative opportunities. The local‐wise worked with stakeholders on visions and pathways for the *collective local youth intelligence*, which then points to a road‐mapping for short‐/medium‐/long‐term action. Some key points here respond to the Lancet Commission recommendations (Lancet Psychiatry, 2024):

- *Reversing intergenerational inequality*: from the decimation of youth clubs in the austerity years, new initiatives point towards young persons' spaces for micro‐business, creative arts, digital development and social enterprise.
- *Improving housing… affordability*: while housing is a national challenge, forward‐looking local providers take on a wider role. Current trends may point to new hybrid housing forms and tenures, ‘short‐social‐rental’ markets and young persons' accommodation platforms.
- *Reversing… commodification/privatization of education*: for the ‘hard to reach’ alternative approaches may have more to offer, and today's bike workshops point towards wider skills empowerment, maybe starting with digital apps, games and content.
- *Improving conditions and rights of younger workers*: local economic policies should extend to employers' compacts, youth skills projects, or social economy activities in household services, local food production, or circular economy repairs.
- *Policies… for the climate emergency*: local greening/food projects may be the best way towards social inclusion and empowerment: dedicated spaces can be managed/owned by young persons, especially for gender, ethnic or cultural minorities.
- *limit the harm caused… by unregulated social media platforms and smartphones*: some progress on school controls; however, the digital landscape moves very fast, with new potential threats such as generative AI. One practical opportunity is to enable young persons' resources and expertise: Here, the Area Youth Forums could provide resources for ‘hackathons’ for local apps and content, for engagement and empowerment in the digital age.

### Next steps

The challenges are huge, but the opportunities and initiatives are many. The Local‐wise model aims to demonstrate and test new ways to enable old relations and synergies of trust, identity and mutual responsibility between young persons and the society around them. The ‘linkages’ framework above, for coordination between education, health, policing, transport, etc., is a means to an end. The key priority is then the ‘lateral’ and ‘citizen’ linkages and synergies, which can enable and empower the many kinds of forums, partnerships, networks, projects and outreaches. The real success will be as far as the young people themselves can shape the next steps.

## Funding information

This project has been supported by the Humanities Strategic Investment Fund of the University of Manchester, NE/S013172/1.

## Conflict of interest statement

The authors have declared that they have no competing or potential conflicts of interest.

## Ethics statement

Obtained approval via the University of Manchester Ethical Decision Tool, date of approval 22‐01‐24.

## Data Availability

Primary data available through www.manchester.ac.uk/synergistics/city‐wise‐local‐wise/.

## Footnotes

^1^

www.manchester.gov.uk/info/200088/statistics_and_intelligence/7611/intelligence_hub.

^2^

https://jobs.manchester.gov.uk/functions/neighbourhoods.aspx.

^3^

www.manchester.ac.uk/synergistics/city‐wise‐local‐wise/.

^4^

www.beeintheloop.co.uk.

^5^

https://childfriendlymanchester.co.uk/manchester‐youth‐council/area‐youth‐forums/.

^6^

https://msmpowerhouse.org.uk/.
